# Gut bacterial metabolism produces neuroactive steroids in pregnant women

**DOI:** 10.1093/lifemeta/loae030

**Published:** 2024-07-19

**Authors:** Kelsey E Huus, Ruth E Ley

**Affiliations:** Department of Microbiome Science, Max Planck Institute for Biology, Tübingen 72076, Germany; Department of Microbiome Science, Max Planck Institute for Biology, Tübingen 72076, Germany

**A multitude of bioactive compounds are produced by the gut microbiota, including metabolites with notable antimicrobial or immunomodulatory effects. Perhaps the most compelling of these compounds, however, are the ones with the potential to affect human mood and behavior. In an article published recently in *Cell,* McCurry *et al.* demonstrated that human gut bacteria are capable of producing neuroactive steroids, including a drug clinically approved by the United States Food and Drug Administration (FDA) for the treatment of postpartum depression.**

**Figure 1:**
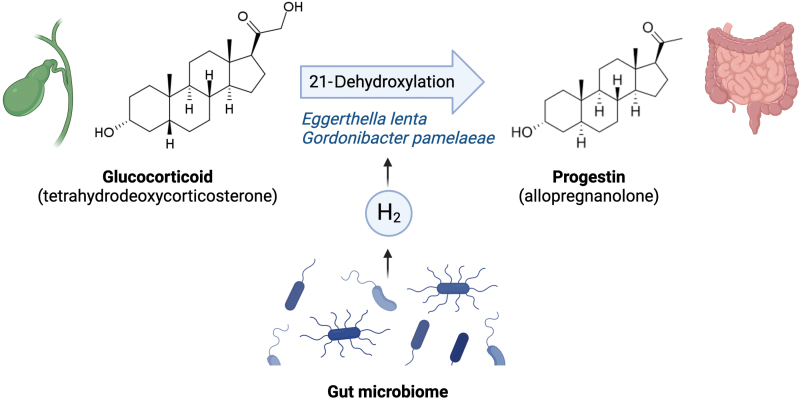
Intestinal bacteria *Eggerthella lenta* and *Gordonibacter pamelaeae* are capable of transforming bile-produced glucocorticoids (e.g., tetrahydrodeoxycorticosterone) into progestins (e.g., allopregnanolone) via 21-dehydroxylation. This process is stimulated in the presence of hydrogen gas produced by other gut bacteria. The figure was created using BioRender.

Steroids are a diverse class of compounds characterized by four fused rings of carbon and include biologically important compounds such as cholesterol and sex hormones. Steroids are also critical in the gut: bile, secreted into the gastrointestinal tract to aid digestion, is mainly composed of cholesterol-derived bile acids and a smattering of other bioactive steroids. Once in the gut, bile-derived compounds are “ripe for the picking” in terms of microbial metabolism, and are subjected to a dizzying array of modifications by gut bacteria. McCurry *et al*. demonstrated that bile-derived glucocorticoids—a class of steroids implicated in glucose metabolism and immune function—can be modified by gut bacteria to produce progestins, a class of sex hormones and neurosteroids (Fig. 1) [1]. These bacterially produced progestins include allopregnanolone, the above-mentioned treatment for postpartum depression.

The specific type of chemical transformation required to convert glucocorticoids to progestins was already suspected to exist in the gut microbiome; however, microbiological and molecular details have been lacking. McCurry *et al*. showed that members of the *Eggerthellaceae* family, in particular *Gordonibacter pamelaeae* and *Eggerthella lenta,* were capable of modifying glucocorticoids into progestins via 21-dehydroxylation *in vitro*. Moreover, the authors were able to identify a four-gene cluster responsible for the enzymatic activity. Although a clean knock-out of the system was not possible (and it is, therefore, unclear whether all four genes were strictly necessary), the authors used homologous expression to demonstrate that the cluster was sufficient to confer 21-dehydroxylation activity in related strains. The abundance of *G. pamelaeae* and *E. lenta,* and of the identified genes correlated with progestin abundance in human feces. It therefore seems highly likely that these players are responsible for *in vivo* production of progestins in the gut.

Microbial activity in communities is rife with co-dependencies, a property that can complicate single-strain *in vitro* studies. This study found that 21-dehydroxylation activity by *Eggerthellaceae* was amplified in the presence of hydrogen-producing helper bacteria such as *Escherichia coli* (Fig. 1). The productivity boost was directly attributable to hydrogen gas, a common end product of microbial fermentation in the gut. Hydrogen is already known to promote other types of metabolic activity within the gut microbiota, such as acetogenesis and methanogenesis (the production of acetate and methane gas, respectively); however, it is interesting that other enzymatic reactions are also affected by this common end product. The impact of gas production on microbial metabolism may therefore be more complex than first appreciated.

Taken more broadly, the implications of a microbially produced postpartum drug are notable, not least because it points to a possible system of gut–brain communication. Production might be particularly relevant during pregnancy: McCurry *et al*. found that fecal progestin levels were much higher in pregnant women compared to nonpregnant women and men. Moreover, feces transplanted from a pregnant woman into germ-free mice were able to confer the production of intestinal progestins *in vivo,* demonstrating a clear role for the human microbiome in this activity. It is therefore tempting to speculate that microbial production of progestins is deliberately increased during pregnancy due to its benefit to the host. However, it is also possible that other hosts or dietary factors are responsible for the increased progestins during pregnancy; the authors did not directly show that the microbiomes of pregnant women had increased progestin synthetic capacity compared to microbiomes of nonpregnant individuals. It is also unclear whether the progestins produced in the gastrointestinal tract can make it into circulation, or to what degree gut-derived compounds at those concentrations can affect host mood or behavior. Therefore, much remains to be uncovered regarding the translational impact of these findings.

In summary, McCurry *et al.* identify a metabolic pathway by which gut microbes transform bioactive steroids in the gut, producing progestins with known neuroactivity. The authors identify both the bacteria and the genes responsible for this enzymatic activity, showing that certain strains of *E. lenta* and relatives are capable of 21-dehydroxylation. This activity is enhanced in the presence of hydrogen gas produced by their microbial neighbors. Moreover, the abundance of these progestins is increased in the gastrointestinal tract of pregnant women, implying a possible modulation of the gut–brain axis with consequences for host health. Nevertheless, more research is required to clarify the clinical implications of these findings and the possible role of bacteria-derived progestins in modulating human behavior.
